# Cellulose–Chitosan Antibacterial Composite Films Prepared from LiBr Solution

**DOI:** 10.3390/polym10101058

**Published:** 2018-09-23

**Authors:** Jiwook Yang, Gu-Joong Kwon, Kyojung Hwang, Dae-Young Kim

**Affiliations:** 1 Department of Biological and Environmental Science, Dongguk University-Ilsan, Biomedical Campus, Goyang-si, Ilsandong-gu 10326, Korea; jwjii0@naver.com (J.Y.); kyojung7@naver.com (K.H.); 2 The Institute of Forest Science, Kangwon National University, Chuncheon Campus, 1 Gangwondaehakgil, Chuncheon-si, Gangwon-do 24341, Korea; gjkwon@kangwon.ac.kr

**Keywords:** cellulose, chitosan, composite, LiBr, antibacterial

## Abstract

Cellulose and chitosan solutions were prepared in 60% LiBr and mixed with a different weight ratio. The washing and drying of the prepared cellulose–chitosan composite films were performed under identical conditions. The color of the liquefied mixtures and films was initially transparent but changed from colorless to brownish yellow depending on the ratio of chitosan in the solution. The cross section observed in the SEM results indicated that the film developed with a higher ratio of chitosan was more robust and possessed greater antibacterial properties. FT-IR analysis of the films showed that hydrogen bonds between cellulose and chitosan in composite films were successfully achieved and retained excellent mechanical properties. The proper ratio of chitosan in the cellulose solution can increase the tensile strength and improve the elongation of the films; however, the E-modulus property was consistently reduced. The antibacterial activity and mechanical properties of the films were greatly improved as the amount of chitosan in the film increased.

## 1. Introduction

Environmental problems such as resource depletion and climate change have emerged as a result of the increase in fossil fuel consumption associated with industrial and technological development. To help solve these problems, biomass materials must be developed that do not use toxic or noxious components and have biodegradable properties [1].

Cellulose, a well-known biomass, has a linear structure of β-(1−4) glycosidic bonds linked to d-glucose units. It also has a crystalline structure because of inter- and intra-molecular hydrogen bonds [2,3,4]. Cellulose is insoluble in water and other typical organics such as NMMO (N-methylmorpholine-*N*-oxide), LiCl/DMAc (N,N-dimethyl acetamide, NaOH/Urea solution, the dimethyl sulfoxide/ tetrabutylammonium fluoride (DMSO/TBAF), and an ionic liquid. Recently, a new aqueous process for cellulose dissolution and regeneration has been developed. First, cellulose is dissolved in the LiBr solution and then regenerated by cooling it down and washing it to remove the LiBr [5,6,7,8,9].

Chitosan, another type of biomass, has a similar chemical structure to cellulose. It is obtained through the N-deacetylation of chitin—the main component of crustaceans such as crabs and shrimp. Chitosan has various properties including biodegradability, biocompatibility, non-toxicity, and antimicrobial activity. Because of these properties, chitosan has been studied and used as a material across many fields, such as biotechnology, pharmaceutics, agriculture, food, cosmetics, and so on [10,11,12,13].

Recently, cellulose–chitosan blending has been studied to overcome the materials’ separate limitations and provide a variety of applications. The blended materials could be obtained from a dissolved polymer solution by various solvent systems including trifluoroacetic acid, NaOH/thiourea, N-methylmorpholine-*N*-oxide (NMMO), ZnCl_2_·3H_2_O, and ionic liquids. The properties of blended materials with cellulose and chitosan are antibacterial, mechanical characteristics, high adsorption capacities, high porosity, metal ions adsorption, barrier properties, etc. [14,15,16,17,18,19].

The purpose of this study was to prepare composite films mixed with cellulose and chitosan; specifically, cellulose and chitosan were dissolved in a 60% LiBr solution. Furthermore, the composite films were characterized by their physicochemical properties and antibacterial activity.

## 2. Materials and Methods 

### 2.1. Materials

Filter paper (Whatman Filter Paper No. 5, Whatman International, Berkhamsted, UK) and chitosan powder (MW 190~310 kDa, deacetylation degree 75~85%, Sigma Aldrich, St. Louis, MO, USA) were used for the cellulose and chitosan samples. Lithium bromide (Samchun, Pure Chemical Co., Ltd., Seoul, South Korea) was used as the cellulose and chitosan solvent.

### 2.2. Preparation of the Cellulose–Chitosan Composite (CCH) Film

For the preparation, 1 wt % of cellulose and 1 wt % of chitosan were added to 60% LiBr solution and stirred at 300 rpm for 5 min. Then, these suspensions were heated 120 °C at for 20 min. After the cellulose and chitosan were entirely dissolved, the solutions were mixed according to cellulose to chitosan weight ratios of 10:0, 9:1, 8:2, and 7:3 and stirred for an additional 10 min. Mixed solutions were cast on a glass mold to manufacture gels of the same size. The glass mold was preheated to 90 °C to prevent the solution from cooling to a gel during casting. The solution became a gel at 70–80 °C. Then, it cooled down to room temperature. Regenerated composite gels were washed with water for 24 h. The CCH film was manufactured by pressing the composite gels with 2.5 kg weights for 30 min and oven drying at 105 ± 1 °C for 24 h. The CCH films were abbreviated to CCH10, 9, 8, and 7 to represent the ratios of cellulose to chitosan, 10:0, 9:1, 8:2, and 7:3, respectively.

### 2.3. Materials Characterization

The morphologies of the cross section of the CCH films were characterized by scanning electron microscopy (EM-30 Mini SEM, COXEM Co., Ltd., Daejeon, South Korea). The infrared spectra of the composite films were measured using the Fourier transform infrared spectrometer (FTIR; Vertex70, Bruker Optics Ltd, Billerica, MA, USA). The mechanical properties—tensile strength, elongation-at-break, E-modulus—of the CCH films were measured using L&W Tensile Tester (AB Lorentzen & Wettre, Kista, Sweden). The film samples were cut into 1.5 × 15 cm for this test.

### 2.4. Antibacterial Evaluation

*Escherichia coli* (KCCM 11234) and *Staphylococcus aureus* (KCCM 12256), obtained from the Korean Culture Center of Microorganisms, were the bacterial strains used in this study. *E. coli* and *S. aureus* were grown in Tryptic Soy Broth (30 g/L, Difco Laboratories Inc., Detroit, MI, USA) for 24 h at 35 °C to test the antibacterial activity of the CCH films. The CCH film samples were prepared and cut into 1.5 × 6 strips. The film samples and 1 mL of the inoculum were added to 1.7-mL microtubes, mixed by vortexing, and incubated for 24 h at 35 °C in a shaking water bath (NEX-703SWO, Nexus Technologies Co., Seoul, South Korea). The inhibitive property of the CCH film was calculated using the dilution plate method.

## 3. Results

### 3.1. Visual Analysis

**Figure 1:**
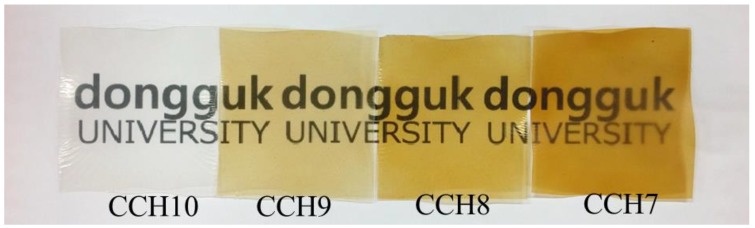
Photo of the CCH films.

The cellulose solution was transparent and the color of the chitosan solution was transparent brown. As the ratio changes from 10:0 to 9:1, 8:2, and 7:3, the solution gradually changed from colorless to brownish yellow. Figure 1 shows photos of the CCH films, which were manufactured from the mixed solution in ratios of 10:0, 9:1, 8:2, and 7:3. 

### 3.2. SEM Analysis

**Figure 2:**
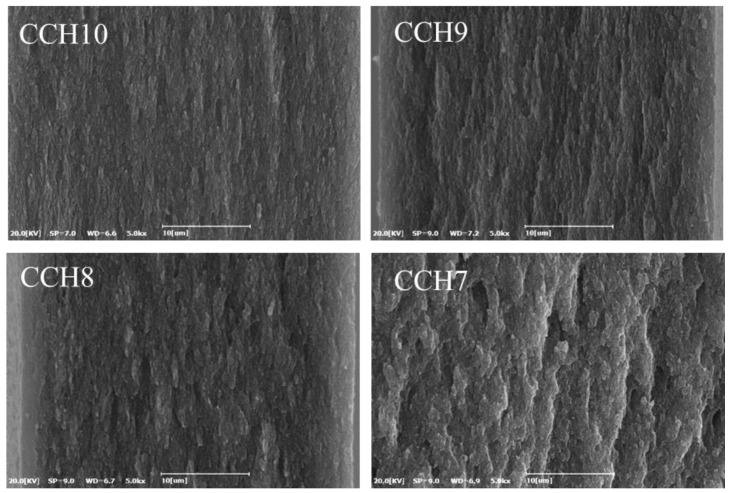
SEM images of the cross section of the CCH films (×5000).

Figure 2 shows SEM images of the cross section of the CCH10, 9, 8, and 7 films. The cross section of CCH10, which contained no chitosan, exhibits a uniform layer structure. CCH9 and CCH8 have a similar layer structure, but they become relatively rougher as the ratio of chitosan increases. CCH7 shows a rough and non-uniform cross section compared to the other films. This is probably because of chitosan agglomeration and phase separation [16,20].

### 3.3. FT-IR Analysis

**Figure 3:**
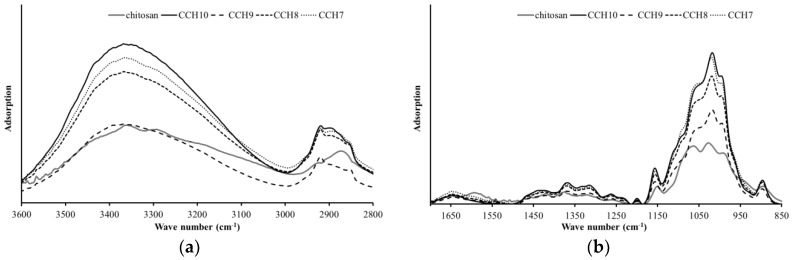
FT-IR spectra of the CCH films in the range of 3600 to 2800 cm^−1^ (**a**) and 1700 to 850 cm^−1^ (**b**).

FT-IR spectroscopy was obtained to analyze the chemical changes of the CCH films as the ratio of chitosan increased. Figure 3 illustrates the FT-IR spectra of the CCH films and chitosan. The FT-IR spectra of chitosan show a typical spectrum of pure chitosan. The peaks at around 894 and 1153 cm^−1^ correspond to a saccharide structure, similar to cellulose. The band at 1336 cm^−1^ is assigned to a vibration of C–H, and 1558 cm^−1^ is assigned to stretching vibration of the amino group of chitosan. The band at 1593 cm^−1^ was related to the NH bending of the amide II. The bands at 1660 and 1323 cm^−1^ have been reported as the amide I and III peaks, respectively. The broad band from 3000 to 3600 cm^−1^ is due to the stretching vibrations of the OH groups, which were overlapped by the stretching vibration of the NH_2_ groups [19,21,22,23,24]. Comparing CCH10, which is only cellulose, with other CCH films, including chitosan, the peak of NH bending vibrations at 1593 cm^−1^ of chitosan increased as the ratio of chitosan increased in the films. In the range from 3000 to 3600 cm^−1^, the peaks at 3292 cm^−1^ disappeared and the 3359 cm^−1^ shifted higher frequency (3367 cm^−1^). This can be attributed to the intermolecular interaction that occurred between the OH and NH groups of cellulose and chitosan during the process of dissolution and regeneration.

### 3.4. Mechanical Analysis

**Figure 4:**
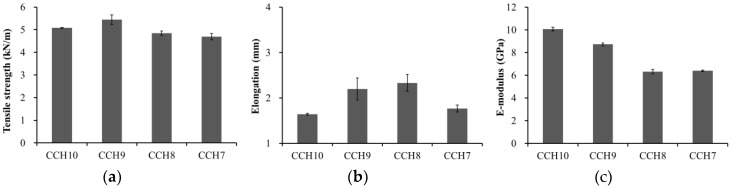
Tensile strength (**a**), elongation (**b**), and E-modulus (**c**) of the CCH films.

Figure 4 shows the tensile strength, elongation, and E-modulus of the composite films across the different ratios of cellulose and chitosan. The tensile strength of the CCH10, 9, 8, and 7 were 5.08, 5.45, 4.84, and 4.69 kN/m, respectively (Figure 4a). The tensile strength increased from CCH10 to CCH9, but the strength of the other films decreased as the ratio of chitosan increased. The elongation of the CCH10, 9, 8, and 7 were 1.64, 2.20, 2.33, and 1.77 mm, respectively (Figure 4b). The elongation increased from CCH10 to CCH8 but rapidly decreased at CCH7. The E-modulus of the CCH10, 9, 8, and 7 were 10.07, 8.73, 6.31, and 6.39 Gpa, respectively (Figure 4c). The E-modulus continuously decreased with an increase of chitosan. It is because the elongation rate is higher than the tensile strength. The increase in tensile strength and elongation can be attributed to a certain amount of chitosan causing an increase in the intermolecular interaction between the –OH group of cellulose and the –OH and –NH_2_ groups of chitosan—this works as a reinforcement for the cellulose matrix. However, the addition of more than a certain amount of chitosan caused tensile strength and elongation decrease. This is because chitosan intramolecular bonds rather than intermolecular bonds with cellulose are formed. It occurs during phase separation between two components, thus causing nonhomogeneous interaction in the film and weakened mechanical properties [16,20,25,26].

### 3.5. Antibacterial Analysis

**Figure 5:**
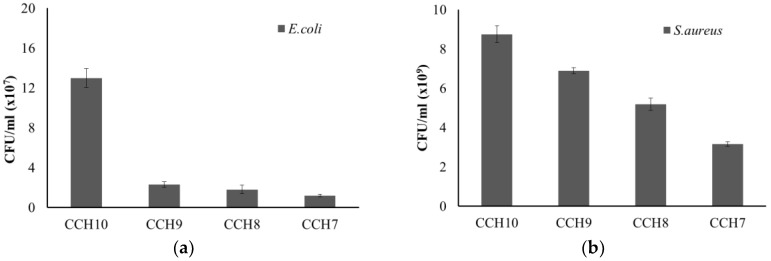
Antibacterial activity of the CCH films on the growth of *E. coli* (**a**) and *S. aureus* (**b**).

Figure 5 shows how *E. coli* and *S. aureus* were used to test the antibacterial activity of the CCH films. The antibacterial activity of both *E. coli* and *S. aureus* gradually increased as the content of chitosan increased in the CCH films. In particular, the CCH films prepared with chitosan profoundly reduced the growth rate of *E. coli*. Many researchers have found numerous antibacterial mechanisms associated with chitosan. One of the most accepted of these is that the positive charges (protonated ammonium group) in chitosan react with negative charges in bacterial cell membranes. This study shows that the chitosan in CCH films exhibited strong antimicrobial activity toward *E. coli* and *S. aureus* [19,27,28,29].

## 4. Conclusions

CCH films were prepared by dissolving cellulose and chitosan in a 60% LiBr solution. As the ratio of chitosan increased, the color of the dissolved mixtures and films gradually changed from colorless to brownish yellow, and the cross section of the films became increasingly robust, with improved mechanical properties. The hydrogen bonds between cellulose and chitosan were formed successfully according to an FT-IR analysis of the films. Additionally, the proper amount of chitosan in the films increased their tensile strength and elongation properties. The antibacterial activity of the composite films was greatly improved as the ratio of chitosan increased. Cellulose–chitosan composite films could serve as a potential biomedical application for biomaterial, medical dressings, and skin tissue engineering.
